# Regulation of EMT in Colorectal Cancer: A Culprit in Metastasis

**DOI:** 10.3390/cancers9120171

**Published:** 2017-12-16

**Authors:** Trung Vu, Pran K. Datta

**Affiliations:** 1 Division of Hematology and Oncology, Department of Medicine, Comprehensive Cancer Center, University of Alabama at Birmingham, Birmingham, AL 35233, USA; ttvu@uab.edu; 2 Birmingham Veterans Affairs Medical Center, Birmingham, AL 35233, USA

**Keywords:** EMT, colorectal cancer, metastasis

## Abstract

Epithelial to mesenchymal transition (EMT) is a process during which cells lose their epithelial characteristics, for instance cell polarity and cell–cell contact, and gain mesenchymal properties, such as increased motility. In colorectal cancer (CRC), EMT is associated with an invasive or metastatic phenotype. In this review, we discuss recent studies exploring novel regulation mechanisms of EMT in CRC, including the identification of new CRC EMT regulators. Upregulation of inducers can promote EMT, leading to increased invasiveness and metastasis in CRC. These inducers can downregulate E-cadherin and upregulate N-cadherin and vimentin (VIM) through modulating EMT-related signaling pathways, for instance WNT/β-catenin and TGF-β, and EMT transcription factors, such as zinc finger E-box binding homeobox 1 (ZEB1) and ZEB2. In addition, several microRNAs (miRNAs), including members of the miR-34 and miR-200 families, are found to target mRNAs of EMT-transcription factors, for example ZEB1, ZEB2, or SNAIL. Downregulation of these miRNAs is associated with distant metastasis and advanced stage tumors. Furthermore, the role of EMT in circulating tumor cells (CTCs) is also discussed. Mesenchymal markers on the surface of EMT CTCs were found to be associated with metastasis and could serve as potential biomarkers for metastasis. Altogether, these studies indicate that EMT is orchestrated by a complicated network, involving regulators of different signaling pathways. Further studies are required to understand the mechanisms underlying EMT in CRC.

## 1. Introduction

Colorectal cancer (CRC) remains the second leading cause of cancer death in the United States. More than 50% of CRC patients will develop liver metastases during their lifespan [1]. Despite the development of treatment regimens, there is no effective therapy for advanced CRC with metastasis. Although inherited genetic susceptibility has an important role in a subset of CRC cases, the vast majority of CRC cases are sporadic and non-inherited [2]. Sporadic pre-neoplastic lesions gradually accumulate genetic and epigenetic modifications that allow uncontrolled proliferation and cell survival, followed by invasive and metastatic properties typical of colorectal carcinoma [3]. Understanding the molecular mechanisms underlying these transitions, especially how colorectal cancer cells acquire invasive and metastatic properties, is important for the development of optimized strategies to treat CRC.

Epithelial to mesenchymal transition (EMT) is a complicated cellular process, during which epithelial cells acquire a mesenchymal phenotype. EMT has been categorized into three types based on the physiological tissue context: (1) embryonic development and organ formation [4]; (2) wound healing and organ fibrosis [5]; and (3) cancer progression [6]. Type 3 EMT is associated with invasive or metastatic phenotype [7]. In the past decade, increasing numbers of studies have provided strong evidence for the vital role that EMT plays in cancer progression and metastasis in many types of malignancies including CRC [8]. During EMT, tumor cells undergo tight junction dissolution, disruption of apical–basal polarity, and reorganization of the cytoskeletal architecture, which enable cells to develop an invasive phenotype. In cancer cells, EMT is abnormally regulated by extracellular stimuli derived from the tumor microenvironment, including growth factors and inflammatory cytokines, along with intra-tumoral physical stresses such as hypoxia [9]. Therefore, EMT programming allows tumor cells to adapt to the constant changes of the human tumor microenvironment, and thus to successfully metastasize. 

In this review, we will describe current knowledge about the mechanisms and regulators of EMT in CRC. These regulators are aberrantly expressed in human CRC and their expression correlates with induction of EMT and cancer metastasis. We will also discuss the importance of studying the role of EMT in metastasis, and how EMT can be considered as an applicable target for therapeutic strategies, especially for patients with CRC metastasis.

## 2. Regulation of EMT in Colorectal Cancer

The process of EMT requires the cooperation, in a timely manner, of a complex network, consisting of molecular signaling pathways and regulators. These factors are categorized into three groups: the effector molecules which execute the EMT program (EMT effectors), the transcription factors which orchestrate the EMT program (EMT core regulators), and the extracellular cues which activate the EMT program (EMT inducers) [10]. 

### 2.1. EMT Effectors

A majority of EMT effectors are subcellular structure proteins that define the epithelial or mesenchymal phenotype of a cell. EMT is characterized by the downregulation of genes encoding for epithelial cell junction proteins (E-cadherin, claudins, and occludins) and the activation of genes, of which the protein products (vimentin, fibronectin and N-cadherin) promote mesenchymal adhesion. Among them, a key feature of EMT is the downregulation of E-cadherin, leading to the destabilization of adherens junctions. In response to a variety of induction signals, E-cadherin is subjected to various levels of regulation, including transcriptional repression [11], promoter methylation [12], and protein phosphorylation and degradation [13]. Loss of E-cadherin expression is associated with a poor prognosis in stage III CRC [14].

Additional key EMT effector molecules are proteins that promote cell migration and invasion during EMT. Fibronectin, an extracellular protein required for mesenchymal cell migration, is frequently induced upon EMT activation. High levels of fibronectin expression are associated with poor prognosis in CRC [15]. Furthermore, knockdown of fibronectin suppresses CRC cell proliferation via the NF-κB/p53-apoptosis signaling pathway and arrested cells in the S phase [16]. Overexpression of N-cadherin, another mesenchymal marker, is correlated with metastasis and worse survival of CRC patients [17]. 

### 2.2. EMT Core Regulators-Transcription Factors Driving EMT

The regulation of EMT requires robust transcriptional machinery, consisting largely of developmental transcription factors which regulate epithelial and mesenchymal markers in a coordinated manner. There are three major groups of EMT-activating transcription factors: the SNAIL family of zinc-finger transcription factors SNAIL/SLUG, the zinc finger E-box binding homeobox (ZEB) family of transcription factors ZEB1/ZEB2, and the TWIST family of basic helix-loop-helix (bHLH) transcription factors TWIST1/TWIST2. The expression of these three groups is activated early in EMT, and thus they have central roles in development and cancer. The roles of these transcription factors in EMT have been well established in a variety of cancers including colorectal cancer. In addition to the three major families of EMT transcription factors, other transcription factors were recently shown to induce EMT in colorectal cancer. One of them is Prospero Homeobox 1 (PROX1) which belongs to the family of homeobox transcription factors. Others are forkhead box (FOX) transcription factors, which are defined by a DNA-binding forkhead domain. These transcription factors contribute to EMT by functionally regulating target genes or controlling the expression of each other. The activities of these EMT transcription factors vary by tissue type and depend on regulatory signaling pathways. Aberrant regulation of EMT-related transcription factors and mesenchymal markers has been identified in CRC and is associated with increased rate of cancer recurrence and decreased survival of CRC patients (Table 1). Previous studies reported that overexpression of EMT-related transcription factors, such as SNAIL [18], SLUG [19], TWIST1 [20], TWIST2 [21], ZEB1 [22], ZEB2 [23] PROX1 [24], FOXC2 [25], FOXQ1 [26], FOXC1 [27], and FOXM1 [28] are associated with invasiveness, metastasis, and poor prognosis of CRC. 

#### 2.2.1. SNAIL Transcription Factors

The SNAIL family of zinc-finger transcription factors, consisting of SNAIL1, SNAIL2, and SNAIL3 (also known as SNAIL, SLUG, and SMUC, respectively), activate the EMT program during development, fibrosis, and cancer [29]. SNAIL promotes EMT primarily through its suppression of E-cadherin. SNAIL binds to E-box DNA sequences of the E-cadherin promoter and recruits histone deacetylases (HDACs) [30], the Polycomb repressive complex 2 (PRC2) [31,32], Lys-specific demethylase 1 (LSD1) [33], G9a [34], and the suppressor of variegation 3–9 homologue 1 (SUV39H1) [35]. This assembly leads to various histone modifications, including methylation and acetylation at histone H3 Lys 4 (H3K4), H3K9, and H3K27, resulting in the suppression of E-cadherin promoter activity. Additionally, SNAIL can induce expression of genes related to the mesenchymal phenotype, such as fibronectin, N-cadherin, and collagen. In addition to regulating epithelial and mesenchymal genes, SNAIL also upregulates other EMT transcription factors, such as SLUG [36], TWIST [37], and ZEB1 [38]. Furthermore, SNAIL can interact with β-catenin and promote the WNT/β-catenin signaling pathway which is also involved in EMT [39]. Therefore, upregulation of SNAIL is considered a major step in EMT. Diverse signaling pathways—NOTCH [40], TGF-β [41], and WNT/β-catenin [42] signaling pathways, cooperate in the initiation and progression of EMT by activating higher levels of SNAIL expression. The critical roles of SNAIL in CRC have been previously determined. In a recent study, SNAIL contributed to the down-regulation of E-cadherin and the vitamin D receptor in colon cancer, leading to the failure of vitamin D analogue treatment [43]. SNAIL is also a critical player in the link between EMT and stem cell properties of CRC, as SNAIL activates the expression of IL-8 by direct binding to its E3/E4 E-boxes, inducing cancer stem cell activities [44].

#### 2.2.2. bHLH Transcription Factors

Basic helix-loop-helix (bHLH) transcription factors are key players in a wide array of developmental processes, including lineage commitment and differentiation. In this family, TWIST1 and TWIST2 are important regulators of EMT. In cancer, TWIST1 was found to repress E-cadherin and induce the expression of mesenchymal markers, such as fibronectin and N-cadherin, during EMT [45]. TWIST1 recruits the methyltransferase SET8, which represses E-cadherin and activates N-cadherin promoter via its H4K20 mono-methylation activity [46]. In CRC, the expression of TWIST1 and TWIST2 is generally restricted to the tumor stroma. Human fibroblast cell lines stably-transfected with TWIST1 acquire characteristics of activated cancer-associated fibroblasts (CAFs) and an increased ability to migrate [47]. TWIST1-positive stromal cells of human CRC have a fully mesenchymal phenotype and are associated with tumor progression [48]. Additionally, a link between epigenetic regulation of TWIST proteins and the tumor-budding phenotype has been reported. The results of laser capture micro-dissection of a high-grade budding case show positive TWIST1 and TWIST2 stroma and no methylation, while a low-grade budding case was characterized by TWIST1 and TWIST2-negative stroma and strong promoter hypermethylation. TWIST1 stromal cell staining was associated with adverse features—more advanced lymph node metastasis, lymphatic vessel invasion, perineural invasion, and worse overall survival. Stromal cells may influence tumor budding in CRCs through expression of TWIST1. Hypermethylation of TWIST1 and TWIST2 promoters in the tumor stroma may represent an alternative mechanism for regulation of TWIST1 [49].

#### 2.2.3. ZEB Transcription Factors

The ZEB family of transcription factors contains two members (ZEB1 and ZEB2), which bind regulatory gene sequences at E-boxes. These factors function as transcriptional repressors and activators, thereby repressing epithelial genes and activating mesenchymal genes, respectively. ZEBs primarily mediate transcriptional repression by recruiting the co-repressor C-terminal-binding protein (CTBP) to E-boxes [50]. In addition, ZEB1 recruits SWitch/sucrose non-fermentable (SWI/SNF) chromatin remodeling protein BRG1 and represses E-cadherin expression [51]. ZEB1 interacts with the transcriptional coactivator p300/CBP-associated factor (PCAF), which switches it from a transcriptional repressor to a transcriptional activator, promoting SMAD signaling [52]. ZEB1 can also recruit LSD1, which likely associates it to histone demethylation in EMT [53]. Interestingly, the upregulation of ZEB2 at the invasive front significantly correlates with tumor stage in primary CRC. Silencing ZEB2 by siRNA decreases the migration and invasion of CRC cells [23]. A recent study showed that that ZEB1 was detected in invasive regions of colorectal tumors and at the tumor–stroma interface: regions that display reduction in polarity components. ZEB1 induces the loss of basement membrane (BM) by suppressing expression of the epithelial BM component Laminin subunit alpha-3 (LAMA3). It binds specifically to Z1, Z2, and E2 on the LAMA3 promoter and represses transcriptional activity [8]. ZEB1 silencing upregulates the expression of several polarity genes in colorectal carcinoma cells, including crumbs homolog 3 (CRB3) and lethal giant larvae 2 (LGL2) [54]. The LGL2 promoter is a direct target of ZEB1 and loss of LGL2 is associated with increased EMT and metastasis in CRC [55]. Moreover, ZEB1 can also promote tumor invasiveness via the regulation of players involved in stroma remodeling: the urokinase plasminogen activator (uPA), and its inhibitor, plasminogen activator inhibitor-1 (PAI-1). ZEB1 binds to the uPA promoter and activates its transcription through a mechanism implicating histone acetyltransferase p300. On the other hand, ZEB1 inhibits plasminogen activator inhibitor-1 PAI-1 expression by reducing the stability of its mRNA [56].

#### 2.2.4. Other Transcription Factors

##### Prospero Homeobox 1 (PROX1)

PROX1 promotes dysplasia in colonic adenomas and CRC progression. PROX1 is upregulated in response to abnormally elevated oncogenic signaling of TCF/β-catenin in intestinal epithelium, important for tumor progression via disruption of cell polarity and adhesion [57]. PROX1 overexpression correlates with increased mesenchymal phenotype, advanced tumor stage, and lymph node metastasis. PROX1 binds to the promoter of pre-miR-9-2 and triggers its expression, resulting in the suppression of E-cadherin 3′-UTR reporter activity and protein expression [58]. 

##### Forkhead Box Q1 (FOXQ1)

FOXQ1 is a member of the Forkhead box transcription factor family. FOXQ1 is overexpressed in epithelial and stromal tumor compartments along with other EMT genes. A recent report demonstrated the upregulation of FOXQ1 in CRC. Ectopic expression of FOXQ1 promoted an anti-apoptotic effect and enhanced tumor growth [59]. FOXQ1 can repress E-cadherin expression by targeting the E-box in its promoter region [60]. FOXQ1 also mediates EMT through the modulation of other EMT-related transcription factors. FOXQ1 interacts with TWIST1 to reinforce the suppression of E-cadherin transcription in CRC [61].

##### Forkhead Box Protein C2 (FOXC2)

FOXC2 is upregulated in human CRC cells and tissues, and correlates with colon cancer progression and patient survival. A functional study demonstrated that FOXC2 promoted cell growth, cell migration, and tumor formation in nude mice, whereas knockdown of FOXC2 significantly reversed these effects. FOXC2 enhances AKT activity with subsequent GSK-3β phosphorylation and SNAIL stabilization. This leads to an induction of EMT and subsequent tumor invasion and metastasis [25]. 

##### Forkhead Box M1 (FOXM1)

FOXM1 has been found to be aberrantly expressed in nearly all carcinomas. FOXM1 stimulates cell proliferation and cell cycle progression by promoting entry into the S-phase and M-phase. FOXM1 is required for proper execution of mitosis. In accordance with its role in stimulating cell proliferation, its expression is regulated by proliferation and anti-proliferation signals, as well as by proto-oncoproteins and tumor suppressors [62]. FOXM1 upregulates the expression of ZEB1/2, and SLUG, consequently leading to a reduction in the expression of E-cadherin [63]. FOXM1D, a novel isoform of FOXM1, has been found to promote CRC EMT and metastasis through activating Rho-associated kinases (ROCKs). ROCKs are known for their pivotal roles in orchestrating actin cytoskeleton, leading to EMT and cancer invasion. The interaction between FOXM1D and ROCK2 is important for the ability of ROCKs to regulate actin arrangement and EMT in a Rho-dependent manner. Furthermore, FOXM1D-induced ROCK activation could be abolished by the ROCK inhibitors Y-27632 and fasudil [64].

### 2.3. EMT Inducers

#### 2.3.1. Signaling Pathways

##### TGF-β

TGF-β signaling plays a dual role in tumorigenesis. It is known to mediate tumor-suppressive effects in the early stages of tumor development. However, in later stages, TGF-β signaling may enhance tumor progression, due to its ability to promote cell proliferation and EMT and to suppress immune function in several cancer types, including those of the breast, prostate, and colon [67]. Among SMADs, SMAD4 has been studied with regard to its involvement in the effects of TGF-β in the later stages of CRC carcinogenesis and in the EMT process. SMAD4 suppresses invasion and restores the epithelial phenotype of SW480 CRC cells. Knockdown of SMAD4-induced levels of endogenous TGF-β cytokines, which leads to increased TGF-β signaling and induction of EMT [68]. SMAD4 is also a negative regulator of STAT3 activation [69]. The loss of SMAD4 leads to aberrant activation of STAT3, which may directly contribute to the EMT process and ZEB1 expression in CRC progression. The loss of SMAD4 causes BMP signaling to switch from tumor-suppressive to pro-metastatic, thereby inducing EMT through activation of RHO and ROCK [70]. Furthermore, activation of TGF-β is important for the EMT-inducing effects of platelets on CRC cells. Upon being secreted from platelets, TGF-β stimulates the expression of EMT markers such as SNAIL, vimentin, and fibronectin in tumor cells [71].

##### WNT/β-Catenin

Over-activation of the WNT/β-catenin pathway promotes EMT-associated dedifferentiation located at the invasive front of colorectal tumors [72]. Recent studies show that both canonical and non-canonical WNT signaling can enhance EMT, depending on the tissue type. Enhanced canonical WNT signaling in CRC cells increases the level of SNAIL, which represses E-cadherin and regulates EMT, thus promoting local invasion [73]. Cytoplasmic SLUG concentration is controlled by GSK3-β phosphorylation and subsequent ubiquitination by β-TrCP. Activation of canonical WNT signaling stabilizes SLUG by inhibiting GSK3β kinase activity and initiates EMT transcriptional programs in cancer cells [74]. WNT3a protein overexpression in CRC patients is concomitant with EMT features, such as reduced expression of the epithelial marker E-cadherin, increased expression of the mesenchymal marker vimentin, and localization of nuclear β-catenin. In vitro and in vivo experiments showed that WNT3a overexpression promotes invasion and induces Snail expression. Dkk1 (an antagonist of WNT/β-catenin signaling) partially reverses the expression of EMT-associated proteins in WNT3a-overexpressing cells [75]. Additionally, the non-canonical WNT pathway is also involved in EMT. There is a high correlation between Fzd2, its ligands WNT5a/b, and EMT markers. It has been shown that Fzd2 expression enhances EMT and cell migration via interaction with STAT3 Stat3. Targeting of Fzd2 by a specific antibody reduces metastasis in a xenograft mouse model of colon cancer [76]. A novel WNT/β-catenin inhibitor IWR-1 suppresses CRC tumor metastasis in relation to EMT [77]. 

#### 2.3.2. Novel EMT Inducers in Colorectal Cancer

**Figure 1:**
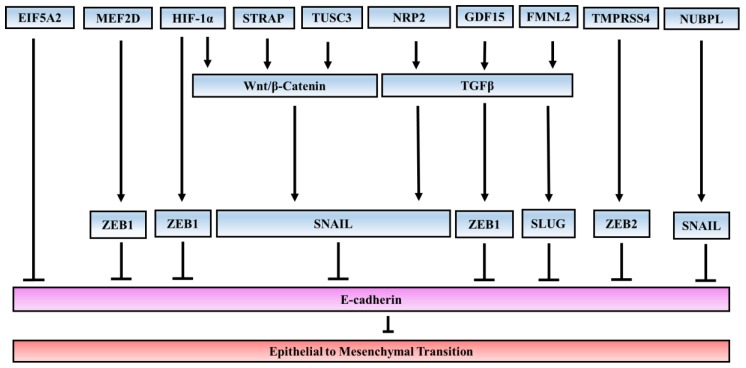
Crosstalk network of novel EMT inducers. Several inducers of EMT in CRC have been identified. These inducers were found to directly regulate expression of EMT-transcriptional factors or do so through other oncogenic signaling pathways. Myocyte enhancer factor 2D (MEF2D), hypoxia-inducible factor 1 alpha (HIF-1α), and transmembrane protease/serine 4 (TMPRSS4) can promote EMT by inducing expression of ZEB transcriptional factors, whereas nucleotide binding protein-like (NUBPL) can upregulate SNAIL. Other proteins such as serine–threonine kinase receptor-associated protein (STRAP) and TUCS3 can induce the WNT/β-Catenin signaling pathway, which has been found to be involved in EMT in CRC. Additionally, neuropilin-2 (NRP2), growth differentiation factor 15 (GDF15), and formin-like2 (FMNL2) can promote TGF-β signaling and induce EMT. Overexpression of eukaryotic initiation factor 5A2 (EIF5A2) can promote EMT through an unknown mechanism which may involve C-Myc and metastasis-associated protein 1 (MTA-1).

In line with the studies on signaling pathways which contribute to EMT, a number of studies have elucidated several novel EMT inducers that also support EMT. These inducers can regulate expression of EMT-TF or activate other signaling pathways which are involved in EMT (Figure 1).

##### Transmembrane Protease/Serine 4 (TMPRSS4)

TMPRSS4 is a member of the type II transmembrane serine protease (TTSP) family and it is highly expressed in CRC tissues and correlates with pathological stages. TMPRSS4 overexpression increases the proliferation and self-renewal ability of CRC cells [78]. Overexpression of TMPRSS4 leads to a significant increase in both in vitro invasion and in vivo metastasis of colon cancer cells. TMPRSS4 induces loss of cell–cell adhesion through E-cadherin downregulation concomitant with the induction in SIP1/ZEB2. TMPRSS4 enhances expression of the integrin subunit α5 [79], which has been centrally implicated in EMT induction and cell motility [80]. Functional blockade of integrin α5β1 demonstrated that this integrin has an important role in TMPRSS4-mediated effects [81]. Blockage of ITG-α5 antibodies (volociximab) is being evaluated in clinical trials for cancer treatment. The possibility of co-targeting both proteins would be an interesting approach to assess synergistic anti-tumor efficacy [82]. In addition, TMPRSS4 overexpression leads to an intracellular signaling cascade that involves FAK, ERK1/2, Akt, Src, and Rac1 activation. Inhibition of PI3K or Src reduces invasiveness and actin rearrangement mediated by TMPRSS4 without restoring E-cadherin expression [83]. A recent study has reported the evaluation of a novel series of 2-hydroxydiarylamide derivatives for the inhibition of TMPRSS4 serine protease activity and the suppression of cancer cell invasion. These derivatives show promising anti-invasive activity of colon cancer cells overexpressing TMPRSS4 [84]. 

##### Formin-Like2 (FMNL2)

FMNL2 is a member of the diaphanous-related formins which act as effectors of Rho family GTPases and control actin-dependent processes such as cell motility/invasion. Overexpression of FMNL2 in metastatic cell lines and tissues of colorectal carcinoma is associated with more aggressive tumor behavior [85]. FMNL2 is involved in mesenchymal phenotype maintenance in human CRC cells. Knockdown of FMNL2 leads to a mesenchymal–epithelial transition confirmed by the upregulation of E-cadherin, α-catenin, and γ-catenin; and downregulation of vimentin, SNAIL, and SLUG. Loss of FMNL2 expression lowers the ability of TGF-β to induce EMT, which suggests that FMNL2 contributes to the acquisition of a mesenchymal and highly migrating phenotype in CRC cells induced by TGF-β. The Ras–MAPK pathway is also involved in FMNL2-induced EMT [86]. Furthermore, Zhu et al. revealed that cytoskeletal regulation by the Rho GTPase pathway, the WNT pathway, the G-protein pathway, and the P53 pathway are affected by FMNL2 [85]. FMNL2 is identified as a target of a number of microRNA in CRC, including mir-206 [87], mir-613 [88], mir-34a [89], and mir-137 [90].

##### EIF5A2

The gene *EIF5A2* encodes eukaryotic initiation factor 5A2 (EIF5A2) and is located on chromosome 3q26, a region frequently amplified in CRC [91]. Ectopic expression of EIF5A2 in CRC cells promotes EMT, cell motility, and invasion in vitro. Overexpression of EIF5A2 is associated with tumor metastasis, determined to be an independent predictor of shortened survival in CRC patients [92]. Overexpression of EIF5A2 in CRC cells enhances the enrichment of c-Myc on the promoter of metastasis-associated protein 1 (MTA1). MTA-1 expression is associated with EMT and metastasis in CRC cells [93]. 

##### Growth Differentiation Factor 15 (GDF15)

GDF15 is a divergent member of the BMP-subfamily of the TGF-β superfamily. GDF15 is also referred to as macrophage inhibitory cytokine-1 (MIC-1), prostate-derived factor (PDF), placental bone morphogenetic protein (PLAB), placental transforming growth factor (PTGF), and nonsteroidal anti-inflammatory drug-activated gene-1 (NAG-1) [94]. GDF15 serves as a negative CRC prognostic marker, and high levels of GDF15, both in tumor tissues and plasma, correlate with an increased risk of recurrence and reduced overall survival [95,96]. It has been considered as a target for CRC therapy [97]. GDF15 promotes CRC cell metastasis both in vitro and in vivo through activating EMT. It binds to TGF-β receptor to activate SMAD2 and SMAD3 pathways. Clinical data shows increased GDF15 levels in tumor tissues and serum, which correlate with reduced CRC overall survival [98]. 

##### Hypoxia-Inducible Factor 1 Alpha (HIF-1α)

It is well recognized that HIF-1α is involved in cancer metastasis, chemotherapy resistance, and poor prognosis. It induces EMT in a variety of cancer types, including those of the colon, breast, lung, head and neck, thyroid, and prostate. HIF-1α expression is independently associated with poor prognosis in CRC by regulating the expression of EMT-related transcription factors [99]. It directly influences ZEB1 expression through the hypoxia response element 3 (HRE-3), located in the ZEB1 proximal promoter. Inhibition of ZEB1 abrogates HIF-1α-induced EMT and cell invasion [100]. HIFs are also involved in EMT through regulating canonical WNT signaling. HIF-1α silencing decreased the stability and transcriptional activity of β-catenin in CRC cell lines [101]. In a recent study, deferoxamine, a hypoxia-mimetic agent, was found to reduce EMT in CRC. Under hypoxia, dexamethasone treatment inhibits HIF-1α protein levels and decreases mRNA levels of hypoxia-induced SNAIL, SLUG, and TWIST1, and transcriptional factors involved in EMT, as well as the hypoxia-induced integrin αVβ6 protein, a well-known EMT marker for CRC cells [102].

##### Myocyte Enhancer Factor 2D (MEF2D)

MEF2D is a transcription factor of the MEF2 family, which was first identified as a muscle gene expression regulator [103]. MEF2 plays central roles in the transmission of extracellular signals to the genome and in the activation of genetic programs that control cell differentiation, proliferation, morphogenesis, survival, and apoptosis of a wide range of cell types [104,105]. MEF2D acts as a developmental transcription factor in embryogenic processes in which EMT is active, such as gastrulation and cardiogenesis. MEF2D is abnormally expressed in human CRC and its upregulation correlates with cancer metastasis. It responds to various tumor microenvironment signals, including not only cytokines such as EGF, IL-6, bFGF, and IGF2, but also the physical stimulus of hypoxia. These microenvironmental factors are often over-activated and contribute to tumor initiation, progression, metastasis, and therapy resistance in cancers including CRC. Upon activation, MEF2D transcriptionally regulates ZEB1 expression. Therefore, MEF2D can function as a central integrator, transducing multiple signals to activate EMT-relevant genes and inducing the metastatic capacity of CRC cells [106].

##### Nucleotide Binding Protein-Like (NUBPL)

NUBPL, also known as IND1 or huInd1, is an assembly factor for human mitochondrial complex I, the largest member of the mitochondrial respiratory chain. NUBPL is significantly overexpressed in CRC tissues compared to normal tissues, and its expression level is positively associated with lymph node metastasis and advanced stage. Overexpression of NUBPL notably promotes the migration and invasive ability of CRC cell lines SW480 and SW620, whereas knockdown of NUBPL leads to the opposite effect. It induces EMT, characterized by downregulation of epithelial marker (E-cadherin) and upregulation of mesenchymal markers (N-cadherin and vimentin). Moreover, it activates ERK signaling, believed to promote EMT and tumor metastasis, as inhibition of ERK suppresses the NUBPL-induced changes in EMT and cell motility [107]. 

##### Neuropilin-2 (NRP2)

Neuropilins (NRPs), initially characterized as cell guidance molecule receptors for semaphorins, act as co-receptors for cancer related growth factors and are involved in several signaling pathways leading to cytoskeletal organization, angiogenesis, and cancer progression. NRP2 (neuropilin-2) confers a fibroblastic shape to cancer cells, suggesting an involvement of NRP2 in EMT. Presence of NRP2 in CRC cell lines is correlated with loss of epithelial markers, such as cytokeratin-20 and E-cadherin, and with acquisition of mesenchymal molecules, such as vimentin. NRP2 is identified as a receptor for TGF-β1. NRP2 expression on CRC cell lines has been shown to promote TGF-β1 signaling, leading to a constitutive phosphorylation of the SMAD2/3 complex. Treatment with specific TGF-β type1 receptor kinase inhibitors restores E-cadherin levels and partially inhibits NRP2-induced vimentin expression, suggesting NRP2 cooperates with TGF-β1 receptor to promote EMT in CRC [108]. Interestingly, NRP2b, a spliced isoform of NRP2, was up-regulated during TGFβ-mediated EMT and knockdown of NRP2b significantly inhibited TGFβ-stimulated EMT and migration [109]. 

##### Tumor Suppressor Candidate 3 (TUSC3)

TUSC3 has been identified as a putative tumor suppressor in a variety of malignancies, including those of the prostate [110], glioblastoma [111], ovaries [112], and pancreas [113]. The TUSC3 protein is localized to the endoplasmic reticulum and is a subunit of the endoplasmic reticulum-bound OST complex, responsible for the N-glycosylation of nascent proteins. The *TUSC3* gene is located in chromosomal region 8p22, a region in which allelic losses are frequent in cancer [114]. In CRC, an association of 8p allelic loss with poor outcome in CRC has been reported. However, recent studies support the oncogenic function of TUSC3 in CRC [115]. TUSC3-overexpressing CRC cells have increased activities of proliferation, invasiveness, and tumorigenesis. TUSC3 is found to be associated with the MAPK and PI3K/Akt signaling pathways. TUSC3 overexpression in CRC cells increases EMT, accompanied by downregulation of E-cadherin and upregulation of vimentin. Furthermore, TUSC3 promotes EMT through induction of WNT/β-catenin signaling pathways. The TUSC3 protein is co-localized with β-catenin, a key component of the WNT signaling pathway through protein–protein interaction [116]. 

##### Serine–Threonine Kinase Receptor-Associated Protein (STRAP)

The multiple roles of STRAP in human cancers have been previously described. Overexpression of STRAP has been reported in lung [117], colon [118], and breast carcinomas [119]. STRAP consists of seven WD40 domains, allowing it to function as a scaffold protein [120]. STRAP was initially identified as a putative inhibitor of the canonical TGF-β signaling pathway [121]. Because the SMAD-dependent TGF-β pathway negatively regulates cellular growth, early studies suggested that STRAP can function as an oncogene. Kashikar et al. showed that loss of STRAP expression induces a mesenchymal-to-epithelial transition through upregulation of E-cadherin, indicating the role of STRAP in EMT [122]. Knockdown of STRAP reduces CRC cell invasion and metastasis in vitro and in vivo. Furthermore, we have observed that STRAP can stabilize β-catenin by inhibiting its ubiquitin-dependent degradation, thus resulting in the inhibition of the expression of its downstream target gene [123]. 

#### 2.3.3. Non-Coding RNA-Mediated Control of EMT 

A number of small non-coding RNAs or microRNAs (miRNAs) regulate the epithelial phenotype and EMT by inhibiting the expression of EMT regulators (Table 2). The expressions of these miRNAs are also regulatory targets of other regulators involved in EMT [124]. 

Members of the miR-200 family (miR-200a, miR-200b, miR-200c, miR-141, and miR-429) are recognized as regulators of the epithelial phenotype through repression of ZEB1 and ZEB2 mRNA translation [125,126,127]. The DNA methylation associated with inactivation of various miR-200 members has been described as a major contributing factor of EMT in cancer. miR-200b and miR-200c transcripts undergo a dynamic epigenetic regulation linked to the EMT or mesenchymal-epithelial-transition (MET) phenotype in tumor progression. The 5′-CpG island hypermethylation-associated silencing of both miR-200 loci is observed in transformed cells with mesenchymal characteristics, including low levels of ZEB1/ZEB2 and high E-cadherin expression [128]. Among miR-200 family members, miR-200c plays a pivotal role in the metastatic behavior of CRC cells. MiR-200c decreases migration and invasion in various CRC cell lines via directly targeting ZEB1 [129]. Methylation-induced downregulation of miR-200c allows upregulation of several of its direct target genes—*ZEB1*, *ETS1*, and *FLT1*. In contrast, hypomethylation of miR-200c mediates the MET process, which results in the settlement of metastasized cells at secondary sites. During MET, hypomethylation-induced re-expression of miR-200c suppresses the EMT-driving genes, accompanied by high E-cadherin and low Vimentin expression [125]. miR-429 reverses TGF-β-induced EMT by interfering with Onecut2 in CRC cells [130]. In addition, ZEB2 is also identified as a direct target of miR-132 [131], miR-192 [132], and miR-335 [133]. Downregulation of these miRNAs is associated with distant metastasis and advanced-stage tumors. 

Members of the miR-34 family are induced by the tumor suppressor p53 and are known to inhibit EMT, and therefore, presumably suppress the early phases of metastasis. MiR-34 members inhibit metastasis formation in CRC via the EMT-regulating network in SNAIL/ZNF281 [134] and the IL-6 receptor (IL-6R)/STAT3 [135]. MiR-34a/b/c targets a conserved seed-matching sequence in the SNAIL 3′-UTR. Overexpression of miR-34a induces mesenchymal-epithelial-transition (MET) and down-regulation of SNAIL. However, suppression of miR-34a/b/c causes up-regulation of SNAIL with the display of EMT markers and related features and enhanced migration/invasion. MiR-34a also suppresses SLUG and ZEB1. Conversely, the transcription factors SNAIL and ZEB1 bind to E-boxes of the miR-34a/b/c promoters, thereby repressing their expression as a part of the EMT program [136]. Ectopic miR-34a prevents TGF-β-induced EMT. The suppression of miR-34a was required for IL-6-induced EMT and invasion. Exposure of human CRC cells to the cytokine IL-6 activates oncogenic STAT3 transcription factor, which directly represses the *MIR34A* gene via a conserved STAT3-binding site in the first intron. Interestingly, the IL-6 receptor (IL-6R), which mediates IL-6-dependent STAT3 activation, is identified as a direct miR-34a target. This IL-6R/STAT3/miR-34a feedback loop is found in primary colorectal tumors [135]. 

Additionally, miR-138 [137], miR-212 [138], miR-30b [139], miR-320a [140], miR-598 [141], miR-4775 [142] , miR-675-5p [143], miR-29b [144], miR-363-3p [145], miR-17 [146], miR-139-5p [147], miR-375 [148], and miR-497 [149] also play pivotal roles in regulating the EMT and CRC metastatic processes.

In addition to controlling the expression of EMT transcription factors and inducers, miRNAs also target genes that help to define the epithelial or mesenchymal phenotype; for example, genes encoding adhesion junction and polarity complex proteins and signaling mediators. MiR-9, transcriptionally induced by PROX1, directly represses E-cadherin expression [27]. Integrin-β4 (ITGβ4), exclusively expressed in polarized epithelial cells, is a novel miR-21 target gene and plays a role in EMT regulation. It is remarkably de-repressed after transient miR-21 silencing and downregulated after miR-21 overexpression [150]. 

Recent studies have also demonstrated major roles of several long non-coding RNAs (lncRNAs) in the regulation of EMT in colorectal cancer. The lncRNA H19 functions as a competing endogenous RNA (ceRNA) for miR-138 and miR-200a to abolish their suppressive effects on mesenchymal marker genes *ZEB1*, *ZEB2*, and *VIM* [151]. BRAF-activated lncRNA (BANCR) induces EMT through a MEK/extracellular signal-regulated kinase-dependent mechanism, as treatment with the MEK inhibitor U0126 can restore the epithelial phenotype in BANCR-overexpressed CRC cells [152]. HOX transcript antisense intergenic RNA (HOTAIR) promotes EMT by downregulating E-cadherin and upregulating vimentin and MMP9 [153]. Other lncRNAs such as long non-coding RNA-activated by TGF-β (lncRNA-ATB) [154], actin filament associated protein 1 antisense RNA1 (AFAP1-AS1) [155], taurine-upregulated gene 1 (TUG1) [156], SPRY4 intronic transcript 1 (SPRY4-IT1) [157], and promoter of CDKN 1A antisense DNA damage-activated RNA (PANDAR) [158] are found to promote EMT and metastasis in CRC through unknown mechanisms. 

## 3. EMT–Metastasis

Metastasis is a multistep process by which tumor cells undergo a sequential series of events to disseminate from their primary site and form secondary tumors in distant tissue [163]. In CRC, loss of the epithelial and gain of the mesenchyme-like phenotype of tumor cells at the invasive front results in increased invasiveness [164]. These changes enable tumor cells to migrate through the extracellular matrix and colonize in the lymph/blood vessels, thereby initiating the first step of the metastatic cascade [165]. 

Recent studies suggest the importance of EMT for circulating tumor cells (CTCs) during the metastatic process [165]. CTCs in blood have been considered recently to be both potential seeds for metastasis and biomarkers for early detection of metastasis [166]. The CTCs of CRC acquire mutations in key genes, such as *KRAS* or *TP53*, that are not identical to those in the corresponding tumor tissue. Gene expression analyses reveal a pronounced upregulation of *CD47* in CTCs as a potential immune-escape mechanism [167]. CTCs which display a mesenchymal phenotype are believed to have increased metastatic potential, [164] with loss of E-cadherin and over-expression of vimentin. Vimentin-positive CTCs displayed higher expression of EMT-related regulators, such as ZEB2, SNAIL, and SLUG. Experimental and clinical data suggest that EMT has an important role in the generation of CTCs. TWIST1 induction dramatically increases CTC numbers in a mouse squamous cell carcinoma tumor model. Successful metastasis may depend on CTCs mesenchymal state maintenance [8]. 

Studies with metastatic CRC patients show that EMT-induced CTCs can be used as a prognostic marker and as an indicator of therapeutic response [168,169]. In a recent study, Satelli et al. used the 84-1 antibody to detect cell-surface vimentin (CSV) on EMT CTCs from blood of patients with metastatic colon cancer. Overexpression of CSV was found in metastatic tumors compared with primary tumors, suggesting that CSV expression is mainly associated with metastasis and could serve as a potential metastatic biomarker [170]. In another study, plastin3 (PLS3) was discovered as an EMT-induced CTC marker that was expressed in peripheral blood from patients with CRC with distant metastasis. PLS3 codes for an actin-bundling protein known to inhibit cofilin-mediated depolymerization of actin fibers. The association between PLS3-positive CTCs and prognosis was particularly strong in patients with Dukes B and Dukes C [171]. 

## 4. Conclusions and Future Perspectives

The process of EMT results from a spectrum of changes and transitions in response to environmental stimuli, dependent on tissue and signaling context. EMT initiation and progression involves a complicated crosstalk among networks of signaling pathways and transcriptional regulators. The hallmark of EMT is the downregulation of E-cadherin, a major epithelial marker, which is regulated by a group of EMT transcription factors. These factors, including the SNAIL, TWIST, and ZEB families, play important roles in both embryogenesis and tumorous settings. Importantly, regulation of these EMT-related transcription factors is associated with invasiveness, metastasis, and poor prognosis of CRC, emphasizing the importance of EMT in tumor progression and metastasis. EMT inducers, including oncogenic signaling pathways, mediate EMT through regulating expression of EMT-TF (transcription factor). In this review, we focused on the current understanding of EMT regulation in CRC with emphasis on the following points:

(1) In addition to well-known signaling pathways which contribute to EMT, there are several novel EMT inducers that also support EMT. These inducers promote EMT by regulating expression of EMT-TF or activate other signaling pathways. Among these inducers, MEF2D and HIF-1α are transcription factors that play central roles in extracellular signal transmission, resulting in the activation of genetic programs that control EMT. The potential of using inhibitors which target these EMT inducers should be considered. Deferoxamine can be used to impair HIF1α function, resulting in the suppression of hypoxia-induced EMT. Additionally, 2-hydroxydiarylamide derivatives that inhibit TMPRSS4 serine protease activity suppress EMT and cell invasion.
(2) A number of microRNAs were found to promote EMT in CRC. These microRNAs have targets which belong to all three groups of EMT-related factors: effectors, transcription factors, and inducers. Among those, miR-200 and miR-34 families target ZEB1, ZEB2, SNAIL, and SLUG transcription factors. Other miRNAs target E-cadherin or integrin-β4. These miRNAs are also targets of other signaling pathways, suggesting the important roles they play in the crosstalk between oncogenic signaling pathways and EMT.
(3) Current studies provide evidence for the important roles CTCs play in the metastatic process [166]. CTCs are considered as both seeds for metastasis and markers for early detection of metastasis. CTCs constitute a heterogeneous population of tumor-derived cells with different phenotypes. One of the most common approaches for isolating CTCs is the epithelial cell adhesion molecule (EpCAM)-based enrichment technique. However, recent studies demonstrate that this technique failed to detect CTC subpopulations that had undergone EMT [172]. Aberrant activation of the EMT program has been implicated in the dispersion of CTCs from primary tumors. EMT endows CTCs with mesenchymal phenotypes, and is an early event in the metastatic process. Thus it is conceivable that EMT marker detection in CTCs may facilitate the early detection of metastases, as well as the assessment of new drug targets in clinical trials. Overexpression of several EMT-induced CTC markers, such as cell-surface vimentin and PLS3 are correlated with metastasis, and can be used as potential metastatic biomarkers.

In conclusion, the loss of epithelial and gain of mesenchyme-like phenotype of the CRC cells is associated with increased invasiveness and metastasis. The observations discussed in this review indicate that EMT is orchestrated by a complex and multifactorial network, involving regulators of different signaling pathways. Further studies are imperative to identifying novel regulators and to understand the mechanisms underlying EMT in CRC. 

## Figures and Tables

**Table 1:** Factors/proteins involved in EMT and their relevance to colorectal cancer.

Factors	Description	Relevance to CRC Cancer	Refs
Transcription factors			
SNAIL	Zinc-finger protein, E-box transcriptional repressor	Expression of SNAIL in the tumor stroma correlated with lower survival of cancer patients and with presence of distant metastasis	[18]
SLUG	Zinc-finger protein, E-box transcriptional repressor	Positive expression of SLUG was significantly associated with Dukes stage and distant metastasis	[19]
TWIST1	bHLH factor	Overexpression in primary CRC was associated with shorter overall survival	[20]
TWIST2	bHLH factor	Upregulation in CRC led to poor prognosis, particularly for patients with stage III and IV	[21]
ZEB1	Zinc-finger protein, E-box transcriptional repressor	High expression of ZEB1 correlated with liver metastasis and poor prognosis in CRC	[22]
ZEB2	Zinc-finger protein, E-box transcriptional repressor	High expression of ZEB2 at the tumor invasion front correlated significantly with worsening prognosis	[23]
PROX1	Prospero homeobox protein	High PROX1 expression was associated with poor grade of tumor differentiation, and in colon cancer patients with less favorable patient outcome	[24]
FOXQ1	Forkhead box transcription factor	High expression of FOXM1 and FOXQ1 emerged as independent prognostic factors in CRC patients	[26]
FOXC2	Forkhead box transcription factor	Overexpression of FOXC2 was associated with poor clinical outcome in human colon cancer	[25]
FOXM1	Forkhead box transcription factor	FOXM1 overexpression was a molecular marker predicting increased metastatic potential of CRC and poorer prognosis	[28]
Factors directly associated with EMT			
E-cadherin	Adhesion glycoprotein	Downregulation of E-cadherin expression indicated worse prognosis in CRC patients	[14,65,66]
N-cadherin	Adhesion glycoprotein	Overexpression was correlated with metastasis and worse survival in CRC patients	[17]
Fibronectin	Adhesion glycoprotein	Results indicated that fibronectin levels increased with the progression of CRC	[15]

Abbreviations: EMT, epithelial to mesenchymal transition; CRC, colorectal cancer; FOXC2, forkhead box C2; PROX1, prospero homeobox 1; ZEB, zinc finger E-box binding homeobox; bHLH: basic helix-loop-helix.

**Table 2:** Micro-RNAs (miRNAs) involved in EMT in colorectal cancer.

Factors	Upstream Regulators	Target Genes	Refs
miRNAs involved in inhibiting EMT			
miR-200 family	P53/ZEB1/SIX1	*ZEB1, ZEB2*	[125,126]
miR-429	P53/ZEB1/SIX1	*HOXA5*	[159]
miR-34a	SNAIL, ZEB1, IL-6/STAT3	*SLUG, ZEB1, IL-6R*	[134,135]
miR-9	N/A	*E-Cadherin*	[58]
miR-21	AP-1/ETS1	*ITGβ4*	[150]
miR-138	N/A	*TWIST2*	[137]
miR-132	N/A	*ZEB2*	[131]
miR-30b	N/A	*SIX1*	[139]
miR-598	N/A	*JAG1/Notch2*	[141]
miR-4775	N/A	*TGFβ*	[142]
miR-363-3p	N/A	*SOX4*	[145]
miR-375	N/A	*SP1*	[148]
miRNAs involved in promoting EMT			
miR-17	N/A	*CYP7B1*	[146]
miR-194	N/A	*MMP-2*	[160]
miR-675-5p	N/A	*DDB2*	[143]
miR-150	WNT/β-catenin	*CREB*	[161]
miR-29a	N/A	*KLF4*	[162]

Abbreviations: SIX1, sineoculis homeobox homolog 1; IL-6R, interleukin 6 receptor; ITGβ4, integrin β4; SOX4, SRY-Box 4; CYP7B1, cytochrome P450 family 7 subfamily B member 1; MMP-2, matrix metallopeptidase 2; DDB2, DNA damage-binding protein 2; CREB, cAMP response element binding; KLF4, kruppel like factor 4.
